# Admixture Effects on Coevolved Metabolic Systems

**DOI:** 10.3389/fgene.2018.00634

**Published:** 2018-12-12

**Authors:** Roxanne R. Zascavage, John V. Planz

**Affiliations:** ^1^Department of Microbiology, Immunology and Genetics, Graduate School of Biomedical Sciences, University of North Texas Health Science Center, Fort Worth, TX, United States; ^2^Department of Criminology and Criminal Justice, University of Texas at Arlington, Arlington, TX, United States

**Keywords:** admixture, cytonuclear, oxidative phosphorylation, mitochondria, coevolution, respiration, metabolism

## Abstract

Oxidative phosphorylation (OXPHOS) is the primary energy generating system in eukaryotic organisms. The complexes within the OXPHOS pathway are of mixed genomic origin. Although most subunit-coding genes are located within the nuclear genome, several genes are coded for in the mitochondrial genome. There is strong evidence to support coadaptation between the two genomes in these OXPHOS gene regions in order to create tight protein interactions necessary for a functional energetics system. In this study, we begin to assess the physiological impact of separating coevolved protein motifs that make up the highly conserved energy production pathway, as we hypothesize that divergent matings will significantly diminish the protein interactions and therefore hinder efficient OXPHOS activity We measured mitochondrial activity in high energy-demanding tissues from six strains of *Mus musculus* with varying degrees of mixed ancestral background. Mice with divergent mitochondrial and nuclear backgrounds consistently yielded lower mitochondrial activity. Bioinformatic analysis of common single nucleotide variants across the nuclear and mitochondrial genomes failed to identify any non-synonymous variants that could account for the energetic differences, suggesting that interpopulational mating between ancestrally distinct groups influences energy production efficiency.

## Introduction

Life requires energy. Within eukaryotic organisms, energy is produced through the oxidative phosphorylation (OXPHOS) pathway, a five complex system. In humans and other bilateral mammals, these complexes consist of ~90 subunits, of which 13 are encoded in the mitochondrial genome and the remaining 77 from the nuclear genome. This bi-genomic formation makes the OXPHOS pathway unique in that it requires the interaction of proteins of differing origins to collectively function (Table 1). More than thirty years of scientific research has provided strong evidence for coadaptation of the two genomes in the gene regions coding for OXPHOS subunits (Goodman, 1982; Rand et al., 2004; Bar-Yaacov et al., 2012; Osada and Akashi, 2012; Sloan et al., 2018).

**Table 1:** Genes that code for the proteins of the oxidative phosphorylation pathway including the number of physical interactions the protein product forms.

**Complex**	**Gene**	**Interactions**	**Complex**	**Gene**	**Interactions**	**Complex**	**Gene**	**Interactions**
I	**ND1**	41	I	Ndufs5L		V	**ATP8**	17
I	**ND2**	12	I	Ndufs6	108	V	**ATP6**	1
I	**ND3**					V	ATP5A1	148
I	**ND4**	26	II	SDHA	104	V	ATP5B	192
I	**ND4L**	1	II	SDHB	105	V	ATP5C1	97
I	**ND5**	27	II	SDHC	13	V	ATP5D	61^*^
I	**ND6**	6	II	SDHD		V	ATP5E	16
I	Ndufa1	5				V	ATP5F1	112
I	Ndufa10	48	III	**Cyt b**	10	V	ATP5G1	9
I	Ndufa11	44	III	Uqcrc1	51	V	ATP5G2	3
I	Ndufa12	101	III	Cyt 1		V	ATP5G3	9
I	Ndufa13	82^*^	III	Uqcrfs1	1	V	ATP5H	46
I	Ndufa2	81	III	Uqcr11	4	V	ATP5J	39
I	Ndufa3	16	III	Uqcrc2	133	V	ATP5J2	38
I	Ndufa4	15	III	Uqcrh	44	V	ATP5K	38
I	Ndufa4L2	14	III	Uqcrb	65	V	ATP5L	61
I	Ndufa5	31	III	Uqcrq	83	V	ATP5O	92
I	Ndufa6	19	III	Uqcr10	15^*^	V	ATP6AP1	33
I	Ndufa7	79				V	ATP6V0A1	81
I	Ndufa8	59	IV	**COX1**	32	V	ATP6V0A2	56
I	Ndufa9	96^*^	IV	**COX2**	43	V	ATP6V0A4	2
I	Ndufab1	24	IV	**COX3**	7	V	ATP6V0B	8
I	Ndufb10	42	IV	COX10	1	V	ATP6V0C	25
I	Ndufb11	68	IV	COX11	8	V	ATP6V0D1	124
I	Ndufb2	1	IV	COX15	13	V	ATP6V0D2	19
I	Ndufb3	22	IV	COX17	26	V	ATP6V0E	10
I	Ndufb4	40	IV	COX4	53	V	ATP6V0E2	1
I	Ndufb5	65	IV	COX5A	49	V	ATP6V1A	105
I	Ndufb6	28	IV	COX5B	42	V	ATP6V1B1	106
I	Ndufb7	37	IV	COX6A1	2	V	ATP6V1B2	125
I	Ndufb8	66	IV	COX6A2	5	V	ATP6V1C1	62
I	Ndufb9	68	IV	COX6B1	33	V	ATP6V1C2	28
I	Ndufc1		IV	COX6B2	2	V	ATP6V1D	61
I	Ndufc2	20	IV	COX6C	11	V	ATP6V1E1	70
I	Ndufs1	104	IV	COX7A1		V	ATP6V1E2	6
I	Ndufs2	66	IV	COX7A2	10	V	ATP6V1F	63
I	Ndufs3	133	IV	COX7A2L	11	V	ATP6V1G1	31
I	Ndufs4	92	IV	COX7B	1	V	ATP6V1G2	5
I	Ndufs5	59	IV	COX7B2	0	V	ATP6V1G3	1
I	Ndufs6	108	IV	COX7C		V	ATP6V1H	50
I	Ndufs7	107	IV	COX8A	2	V	Gm10175	
I	Ndufs8	64	IV	COX8B	0	V	Tcirg1	20
I	Ndufv1	99	IV	COX8C	0	V	ATP5OmtL	
I	Ndufv2	70	IV	COX7CmtL				
I	Ndufv3	46	IV	COX5BmtL				
I	Ndufc2L							

Genes are separated by the complex in which they are involved. Genes located within the mitochondrial genome are indicated in bold. Genes with no published interactions are left blank. Genes with both physical and genetic interactions are indicated by

* *. Table created using information obtained from BioGRID 3.4 (Chatr-aryamontri et al., 2017)*.

Changes in amino composition may not only alter the function of a protein, but also affect protein-protein interactions (Grossman et al., 2004). Studies have shown that disassociation of co-adapted genes can create incompatibility between the two genomes, leading to a breakdown in energy production (King, 1989; Barrientos et al., 1998; Gershoni et al., 2010; Correa et al., 2012; Latorre-Pellicer et al., 2016). Experimental approaches that combined nuclear and mitochondrial backgrounds from closely related species have demonstrated this decrease in energy production (Barrientos et al., 1998; Sackton et al., 2003; Ellison and Burton, 2006; Ellison et al., 2008; Niehuis et al., 2008; Meiklejohn et al., 2013). Frequently, the results of these matings include male infertility (Connallon et al., 2018) and numerous publications analyzing hybrid inviability in mice provide indications pointing toward cytonuclear incompatibility (Takeda et al., 2000; Mihola et al., 2009; Latorre-Pellicer et al., 2016). Although there has been considerable focus in disease studies on the role of OXPHOS and individually, the mitochondrial or nuclear genes involved in the pathway, little attention has been given to the interaction of the genomes with regard to energy production and disease susceptibility (Gómez-Durán et al., 2010; Tranah, 2011; Tranah et al., 2011). As Latorre-Pellicer et al. pointed out, technology is advancing to the point that mitochondrial replacement therapy is being utilized during *in vitro* fertilization, but without complete knowledge of the impact the nuclear background has on mitochondrial function. Without analyzing all aspects of the mammalian OXPHOS pathway simultaneously, linking genetics to energetics, a complete understanding of the intricacies involved in energy production and its role in human disease will never fully be understood.

In the present day, population boundaries that limited human interactions are diminishing, creating an environment wherein disassociation of geographically-aligned nuclear and mitochondrial genomes is becoming increasingly inevitable. Here, we assess the impact of such disassociation in high energy-demanding tissues extracted from wild derived strains of the House Mouse, *Mus musculus*. We identified a clear decrease in oxygen consumption levels associated with divergent nuclear and mitochondrial ancestries. We also evaluated the genomes of each mouse for polymorphisms among the nuclear and mitochondrial OXPHOS gene regions and determined there were no obvious genetic variations that could explain the energetic fluctuation. Based on the results presented here, as well as the literature discussed within surrounding such variances, we theorize that the decrease in OXPHOS efficiency is likely due to incompatibilities between the nuclear and mitochondrial protein isoforms or regulatory regions that have become disjunct as a result of admixture.

This study evaluates the association of genotypes with phenotypes on controlled populations of mammals and adds to our understanding of coadaptation effects on energy production in a time in which populations are becoming increasingly admixed. Our findings provide a scientific foundation that can be incorporated into energetics and health disparities studies involving OXPHOS, as well as advancements and utilization in aging studies and general evolutionary process analysis.

## Methods

### *Mus musculus* Sample Selection

In order to establish admixture group standards, genetically distinct subspecies of *Mus musculus* were used as a model in this study. In late 2011, Yang et al. published findings on the genetic diversity in laboratory mouse strains, outlining the phylogenetic origin of common wild-derived and inbred laboratory mouse strains as compared to wild-caught mice (Yang et al., 2011). Based on their characterization of subspecific origin, wild-derived laboratory strains of *M. musculus* representing a broad sampling of the three most common subspecies, *M. m. domesticus, M. m. castaneus*, and *M*. *m. musculus*, were selected for analysis. The phylogenetic origins across the entire genome of 62 wild-derived strains were mapped. Of these, only 26 strains are commercially available. The 26 strains were pared down to 6 that possessed the desired genetic attributes and were available as live specimens. All mice were obtained from Jackson Laboratories (Bar Harbor, ME).

Strains RBF/DnJ and CZECHII/EiJ were chosen as no admixture samples; >98% of the nuclear genome of each strain traces back to a single subspecific origin, which is consistent with the mitochondrial origin. Within the nuclear OXPHOS genes and mitochondria, they contain no ancestral variation. RBF/DnJ is a pure *M. m. domesticus* strain and CZECHII/EiJ is a pure *M. m. musculus* strain. The strains CALB/RkJ and MOLF/EiJ were selected as representatives of a nuclear admixed group; both strains resonate to a primary subspecies that makes up ~75% of their nuclear genomes and matches the subspecific origin of the mitochondria. CALB/RkJ is a *M. m. domesticus* dominant strain with *M. m. castaneus* making up ~21% of its nuclear OXPHOS background. MOLF/EiJ is a *M. m. musculus* dominant strain with *M. m. castaneus* making up ~19% of its nuclear OXPHOS ancestry. The final strains, MOLG/DnJ and SKIVE/EiJ made up a cytonuclear admixed group because the primary subspecies association of the nuclear genome is different than that of their mitochondria. MOLG/DnJ mice are primarily a *M. m. musculus/M. m. castaneus* nuclear OXPHOS hybrid, with *M. m. musculus* accounting for 77% of their nuclear OXPHOS ancestry, however, their mitochondria is of *M. m. domesticus* origin. SKIVE/EiJ is almost a pure *M. m. musculus* nuclear OXPHOS strain, yet their mitochondria are of *M. m. domesticus* origin (Table 2). Matings are expected to blend the entire genome, not just the OXPHOS genes, and therefore origin of the OXPHOS genes specifically for each strain was not considering in admixture grouping, but instead of the entire genome. However, ancestral background for each OXPHOS gene for each utilized strain has been recorded in Supplementary Table 1, and displays ancestral distributions similar to those of the entire genome for each strain.

**Table 2:** The nuclear and mitochondrial backgrounds for the samples used in the study.

**Group**	**Strain**	Nuclear ancestry^*^			**Mitochondrial ancestry**
		**% *domesticus***	**% *musculus***	**% *castaneus***	
No Admixture	RBF/DnJ	**100**	0	0	*domesticus*
	CZECHII/EiJ	0	**100**	0	*musculus*
Nuclear Admixture	CALB/RkJ	**76.523**	2.608	20.869	*domesticus*
	MOLF/EiJ	5.983	**75.214**	18.803	*musculus*
Cytonuclear Admixture	MOLG/DnJ	5.983	**76.923**	17.094	*domesticus*
	SKIVE/EiJ	1.754	**98.246**	0	*domesticus*

*Strains are grouped according to degree of admixture present. Primary nuclear associations are indicated by bold typeface. Highlighted results indicate primary nuclear associations that deviate from the mitochondrial origin*.

* *Nuclear Ancestry was determined across the entire genome. Supplementary Table 1 indicates the ancestry for each OXPHOS gene*.

### Tissue Extraction

Cardiac and hepatic tissues from each mouse were prepared using a previously published protocol (Dunham-Snary et al., 2014), adapted for our tissue types. The Institutional Animal Care and Use Committee of the University of North Texas Health Science Center approved all animal procedures (Protocol 2014/15-33-A04); all experiments were performed in accordance with relevant guidelines and regulations. Prior to tissue extraction, adult male mice were anesthetized with isoflurane in a drop jar and euthanized by decapitation. The heart and liver were removed and placed in a petri dish containing DMEM with 25 mM glucose and 25 mM HEPES (“prep media”) warmed in a water bath to 37°C. Tissue was gently agitated to remove any blood and fur and placed in a clean petri dish with fresh prep media. The left ventricle of the heart and the right lobe of the liver were cut into ~2 mm cubes using a sterilized razor blade, placed into fresh prep media and kept at 37°C in a non-CO_2_ incubator until all samples were prepared for analysis.

### Preparation of Assay Plate

Seahorse XF24 Islet Capture Microplates were used for the bioenergetics analysis and prepared following protocol modifications published in 2014 (Dunham-Snary et al., 2014). Capture microplate screens were soaked in “running media” made from DMEM with 25 mM glucose warmed to 37°C. Each well was filled with 450 μL of warm prep media. Two 2 mm cubes of tissue were placed in each well using sterilized forceps and the pre-wetted screen was then snapped into place on top of the tissue. The tissue was then washed twice with 450 μL of fresh prep media and once with 450 μL of running media. This wash process involved removing old media, adding fresh, and repeating. Prior to analysis, 450 μL of running media was added to each well and the plate was placed in a 37°C non-CO_2_ incubator for 1 h to allow the tissues to equilibrate.

Liver and heart tissues from each experimental mouse were prepared in triplicate with individual replicates run in separate wells, each replicate containing two 2 mm^3^ pieces. In order to eliminate any plate to plate bias, all samples to be evaluated together were placed on a single plate; cardiac samples from one representative of each different strain of mouse were analyzed simultaneously on a single plate, and hepatic samples from one representative of each different strain of mouse were analyzed simultaneously on a second plate. Four wells on each plate were left blank (without tissue) to provide background correction. Each experiment was performed in duplicate, e.g., two identical cardiac tissue plates and two identical hepatic tissue plates were assessed. In total, experimentation included two strains per admixture group, two mice per strain, and three tissue replicates from two separate tissues evaluated from each individual mouse.

### Bioenergetic Analysis

Basal respiration levels were measured on the Seahorse XF24 Extracellular Flux Analyzer following a modified version of the protocol supplied by Seahorse Biosciences (Ferrick et al., 2008). All wells of the extracellular flux assay plate were filled with 1 mL of the supplied calibration buffer and incubated at 37°C without CO_2_ for a minimum of 5 h, but not longer than 18 hours/overnight. Following the calibration and equilibration period, respiration rates were measured 5 times over a 40-min time period using the following protocol: mix the sample for 3 min, wait for 2 min, and continuous oxygen consumption rate (OCR) measurement for 4 min in picomoles per minute.

### Statistical Analysis

Five oxygen consumption readings (pmoles/min) from each well for 120 individual measurements per admixture group were used in the statistical analysis. The measurements were grouped according to admixture level and tissue type, and each grouping was tested for normality. All three groups were determined to have a normal distribution (*p* > 0.05) based on kurtosis and skewness evaluated using the d'Agostino-Pearson test. Single-factor analysis of variance (ANOVA, α = 0.05) was performed for each tissue to determine if there was any statistically significant difference between the three admixture groups. A Tukey Honest Significant Difference (HSD) *post hoc* pair-wise comparison was performed on the ANOVA results to determine how the variance was apportioned. An additional single-factor analysis of variance (ANOVA, α = 0.05) was performed on the four strains of *M. musculus* containing predominantly *M.m. musculus* nuclear background for each tissue to determine if the nuclear background could have been a contributing factor to the observed results.

### Single Nucleotide Polymorphism Selection and Analysis

Yang et al. mapped the phylogenetic origin of the various mouse strains we used in this study using 549,683 SNPs spanning all chromosomes, including X and Y (Yang et al., 2011). From their database, we selected 450 SNPs located within the gene regions that code for OXPHOS subunit proteins. Only SNPs with both variants observed among our strains of interest were used for the study; elimination of SNPs in which the same allele was present across the six strains of mice tested reduced the panel to 289 SNPs. While we acknowledge this is not an exhaustive list of genes involved in energy production, as there are many regulatory genes and tRNAs, etc to consider, we decided to focus our genetic analysis solely on the genetic regions known to be involved in OXPHOS complex subunit formation. Additional studies need to be performed on other contributing gene regions to fully assess the possibility of a non-cyto-nuclear genetic contribution to our results.

The OXPHOS gene region SNP dataset was analyzed using the Ensembl Variant Effect Predictor (VeP) Software (McLaren et al., 2016). The software provides general information regarding the expected change as well as a Sorts Intolerant From Tolerant (SIFT) score. Using the VeP software for initial evaluation of the presumptive effects and effect locations for each of the 289 selected SNPs, we created a final list of six potential OXPHOS-impacting SNPs to be interrogated further; each of the six SNP variants is present in at least one *M. musculus* strain of interest and results in a non-synonymous substitution in the coding region of an OXPHOS-gene. Further analysis of the six SNPs identified included identification of the SIFT score from the VeP Software to determine severity of the instigated changes from the variant, as well as thorough evaluation of the function of the affected protein and any overlap in the function with other genes or proteins.

## Results

### Cytonuclear Admixture Levels Result in a Significant Drop in Basal Respiration Rate

**Figure 1:**
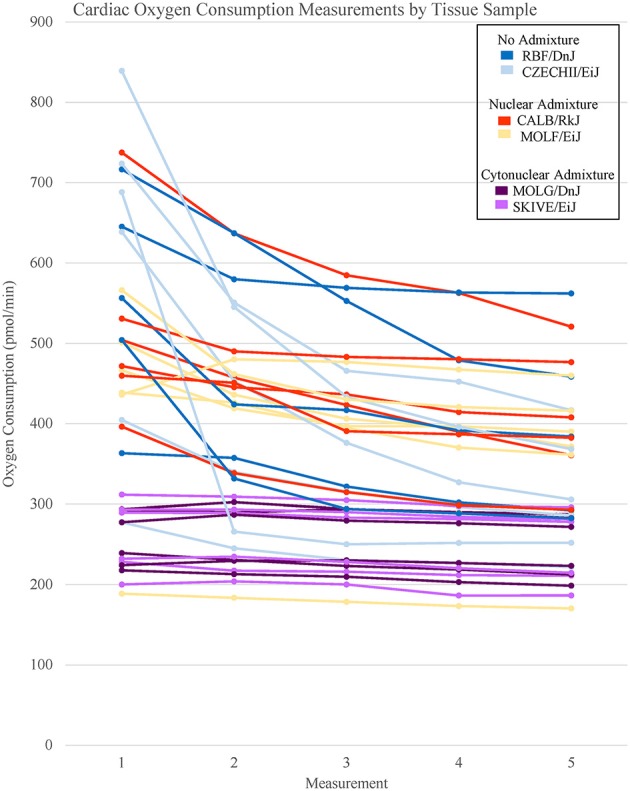
Basal respiration rates of cardiac tissue samples from *Mus musculus* ssp. were analyzed and the oxygen consumption rates were plotted in a line graph. Each line represents a single tissue replicate consisting of five consecutive oxygen consumption measurements. Tissue samples from a single strain of mouse were categorized by color. In total, there are six tissue replicates/lines per strain, three from a single mouse and three from an additional mouse. Cardiac oxygen consumption measurements organized by mouse replicate can be found in Supplementary Figure 1.

**Figure 2:**
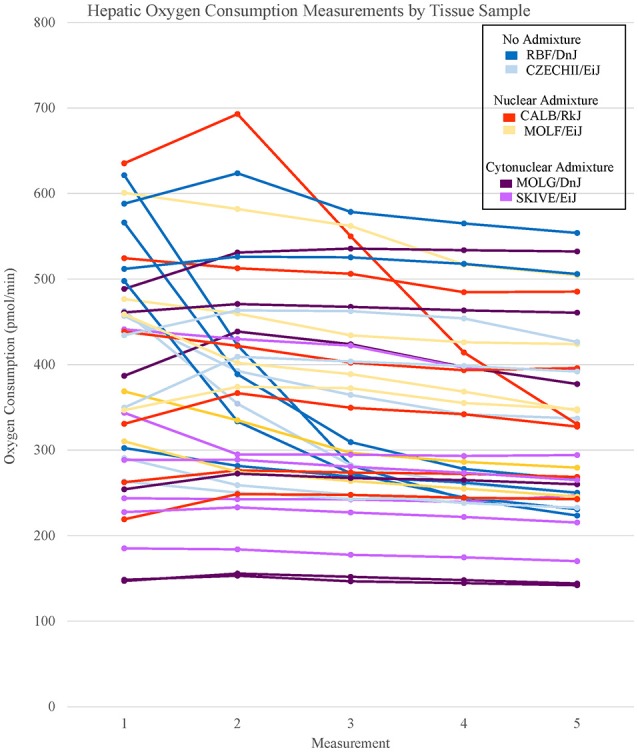
Basal respiration rates of hepatic tissue samples from *Mus musculus* ssp. were analyzed and the oxygen consumption rates were plotted in a line graph. Each line represents a single tissue replicate consisting of five consecutive oxygen consumption measurements. Tissue samples from a single strain of mouse were categorized by color. In total, there are six tissue replicates/lines per strain, three from a single mouse and three from an additional mouse. Hepatic oxygen consumption measurements organized by mouse replicate can be found in Supplementary Figure 2.

**Figure 3:**
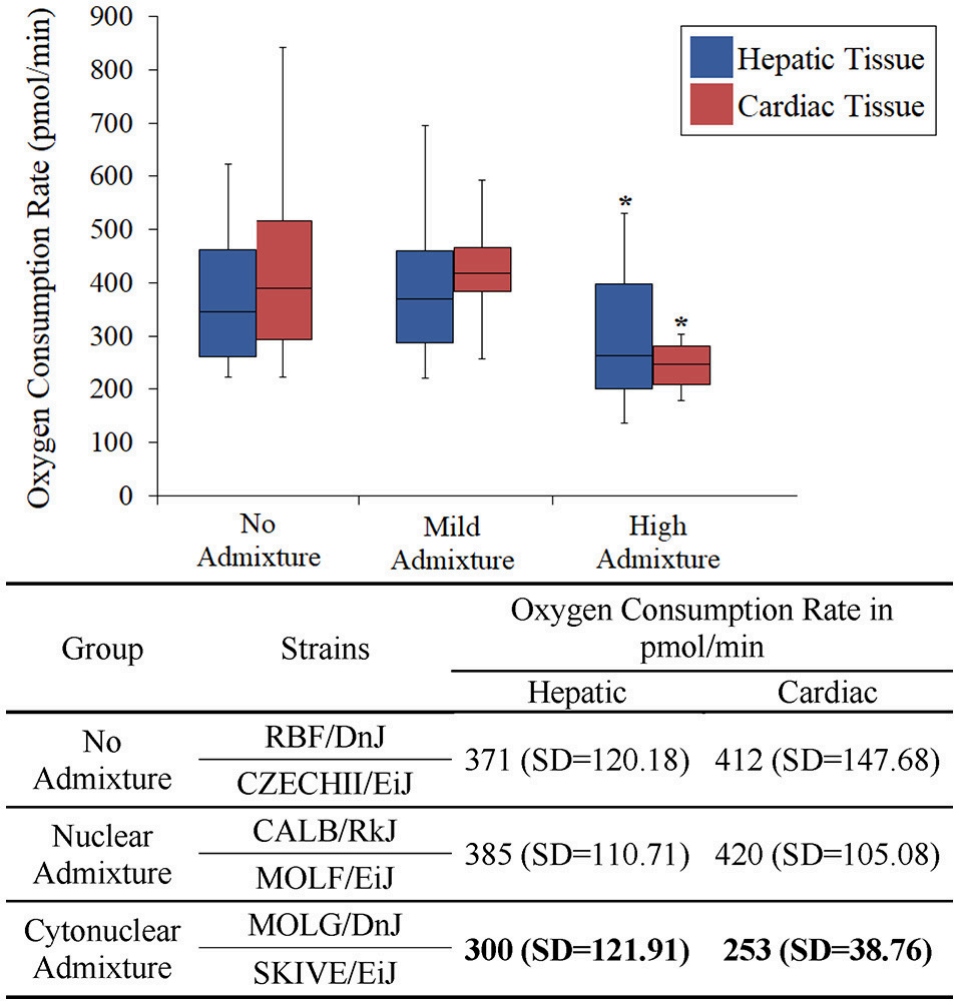
Basal respiration rates of hepatic and cardiac tissue samples from *Mus musculus* ssp. were analyzed and the oxygen consumption rates were plotted in a box plot and whisker diagram. Samples were grouped according to subspecies background concordance between the nuclear and mitochondrial genomes. SD = standard deviation. OCR depicted in the chart represent the average Oxygen Consumption Rate for each group. Significant differences in a group compared to the others is indicated by ^*^. OCR values were significantly lower in the cytonuclear admixture groupings than the other two groups as determined by a one-way ANOVA and Tukey *post-hoc* analysis using an n = 60 (Table 3). Individual OCR results can be found in Supplementary Table 2.

The heart and the liver were selected for bioenergetics analysis due to their high energy demands (Wang et al., 2010). Due to varying metabolic activity, the results from the tissue analyses were not combined; heart results were analyzed separately from the liver results. Oxygen consumption readings for each individual tissue replicate are showing in Figures 1, 2. The trend between admixture groupings was similar in both tissues, as shown in the summary Figure 3. The ANOVA results determined there was no statistical difference between the OCR in the mild-admixture groups and the no-admixture groups in either the hepatic or the cardiac tissues (*p* > 0.05). For both tissues, the cytonuclear admixture grouping demonstrated a significantly lower oxygen consumption level (*p* < 0.01) than the mild- and no-admixture groups (Table 3). Individual OCR read results are listed in Supplementary Table 2.

Table 3A one-way analysis of variance was performed for energetics measurement comparisons between admixture groups.

**Table:** 

**SUMMARY DATA**				
**Population**	**Count**	**OCR sum**	**OCR average**	**OCR variance**
	**Liver | Heart**	**Liver | Heart**	**Liver | Heart**	**Liver | Heart**
No admixture	60 | 60	22,249.28 | 24,720.36	370.82 | 412.01	14,449.33 | 21,806.19
Mild Admixture	60 | 60	23,122.35 | 25,204.24	385.37 | 420.07	12,258.53 | 11,039.54
High Admixture	60 | 60	18,001.47 | 15,182.06	300.02 | 253.03	14,858.20 | 1,500.85

**Table:** 

**ANOVA**						
**Source of variation**	**Sum of squares**	**Degrees of freedom**	**Mean squares**	**F-statistic**	***P*****-value**	**F-crit**
	**Liver | Heart**	**Liver | Heart**	**Liver | Heart**	**Liver | Heart**	**Liver | Heart**	**Liver | Heart**
Between Groups	250,164.92 | 1,064,763.03	2 | 2	125,082.46 | 532,381.52	9.03 | 46.50	**1.85E-5 | 5.88E-17**	3.047 | 3.047
Within Groups	2,452,397.37 | 2,026,447.91	177 | 177	13,855.35 | 11,448.86			
Total	2,702,562.29 | 3,091,210.95	179 | 179

**Table:** 

***post-hoc*** **TUKEY HSD**				
**Group comparison**	**Mean difference**	**95% confidence interval**	**T-value**	**P-value**
	**Liver | Heart**	**Liver | Heart**	**Liver | Heart**	**Liver | Heart**
No to Nuclear	14.517 | 8.065	−37.42-66.46 | −39.15-55.28	0.0676 | 0.413	>0.05 | >0.05
No to Cytonuclear	85.383 | 158.972	33.44-137.32 | 111.76-206.19	3.973 | 8.138	** < 0.01 | < 0.01**
Nuclear to Cytonuclear	70.866 | 167.037	18.93-122.81 | 119.82-214.25	3.298 | 8.55	** < 0.01 | < 0.01**

*Calculation parameters included admixture group measurements with n = 60 (df = 2). The significant p-value confirmed that variance existed between (indicated by bold typeface). A post hoc Tukey HSD test compared the group means using mean square error. Significant results are indicated by bold typeface. These results reveal that the highly admixed hepatic and cardiac samples have respiration levels significantly lower than the other groups*.

Due to the possibility of background bias, the four strains containing *M. m. musculus* nuclear backgrounds, CZECHII/EiJ, MOLF/DnJ, MOLF/EiJ, and SKIVE/EiJ, were compared to confirm the observed results were due to cytonuclear incompatibility. The ANOVA results determined there was a statistical difference between the OCR in the four strains containing *M. m. musculus* nuclear backgrounds (*p* < 0.01) (Table 4). In the hepatic tissues, the cyto-nuclear admixed strain SKIVE/EiJ had oxygen consumption levels significantly lower than those of the nuclear admixed strain MOLF/EiJ. No other significant differences were noted. In the cardiac tissues, the two cyto-nuclear admixture strains, MOLG/DnJ and SKIVE/EiJ displayed significantly lower oxygen consumption levels as compared to the toher two strains, pure ancestry strain CZECHII/EiJ and nuclear admixed MOLF/EiJ. There was no statistical difference between MOLG/DnJ and SKIVE/EiJ (cyto-nulear admixed), nor between CZECHII/EiJ and MOLF/EiJ. Individual OCR read results are listed in Supplementary Table 2 and highlighted in Supplementary Figures 3, 4.

Table 4A one-way analysis of variance was performed for energetics measurement comparisons between all strains containing predominantly *M.m.musculus* nuclear background.

**Table:** 

**SUMMARY DATA**				
**Strain**	**Count**	**OCR sum**	**OCR average**	**OCR variance**
	**Liver | Heart**	**Liver | Heart**	**Liver | Heart**	**Liver | Heart**
CZECHII/EiJ	30 | 30	10,207.28 | 11,844.89	340.24 | 394.83	7,103.19 | 26,065.29
MOLF/EiJ	30 | 30	11,661.99 | 11,678.97	388.73 | 389.30	9,625.68 | 10,964.23
MOLG/DnJ	30 | 30	9,768.56 | 7,607.52	325.62 | 253.58	22,891.61 | 1,257.62
SKIVE/EiJ	30 | 30	8,232.90 | 7,574.54	274.43 | 252.48	5,981.82 | 1,795.22

**Table:** 

**ANOVA**						
**Source of variation**	**Sum of squares**	**Degrees of freedom**	**Mean squares**	**F-statistic**	**P-value**	**F-crit**
	**Liver | Heart**	**Liver | Heart**	**Liver | Heart**	**Liver | Heart**	**Liver | Heart**	**Liver | Heart**
Between Groups	199,240.25 | 580,356.87	3 | 3	66,413.42 | 193,452.29	5.83 | 19.31	**9.6E-4 | 3.19E-10**	2.683 |2.683
Within Groups	1,322,466.66 | 1,162,388.29	116 | 116	11,400.57 | 10,020.59			
Total	1,521,706.91 | 1,742,745.16	119 | 119

**Table:** 

***post-hoc*** **TUKEY HSD**				
**Strain comparison**	**Mean difference**	**95% confidence interval**	***T*****-value**	***P*****-value**
	**Liver | Heart**	**Liver | Heart**	**Liver | Heart**	**Liver | Heart**
CZECH to MOLF	48.49 | 5.53	−122.49-25.51 | −77.68–88.74	1.759 | 0.214	>0.05 | >0.05
CZECH to MOLG	14.62 | 141.25	−59.38-88.62 | 58.04–224.46	0.530 | 5.465	>0.05 |** < 0.01**
CZECH to SKIVE	65.81 | 142.35	−8.19-139.81 | 59.14–225.56	2.387 | 5.508	>0.05 | ** < 0.01**
MOLF to MOLG	63.11 | 135.72	−10.89-137.11 | 52.51–218.93	2.289 | 5.251	>0.05 | ** < 0.01**
MOLF to SKIVE	114.30 | 136.82	40.30-188.30 | 53.61–220.03	4.146 | 5.294	** < 0.01 | < 0.01**
MOLG to SKIVE	51.19 | 1.10	−22.81-125.19 |−82.11–84.31	1.857 | 0.043	>0.05 | >0.05

*Calculation parameters included admixture group measurements with n = 30 (df = 3). The significant p-value confirmed that variance existed between (indicated by bold typeface). A post hoc Tukey HSD test compared the group means using mean square error. Significant results are indicated by bold typeface. These results reveal that the strains of mice with the same nuclear ancestry have respiration levels significantly different from each other*.

### Observed Single Nucleotide Polymorphisms Did Not Explain OXPHOS Variances

In order to eliminate the possibility of a non-cytonuclear incompatibility related genetic defect leading to the loss of energetics efficiency identified in highly admixed strains of mice, a SNP panel spanning the OXPHOS gene regions and the mitochondria was evaluated for any evidence of variations. Table 5 provides the genotypes of the *Mus* ssp. strains across the six SNPs identified in the data parsing process detailed previously. Initial evaluation of the distribution of the missense variants shows no significant trend. One would expect that if a genetic variant was the cause of the observed energetic decline, that the causative variant would be present in the affected strains and not any of the unaffected strains. However, there are no variants present in the cytonuclear admixture samples that are not also represented by at least one mouse strain of a different admixture groups. Assessing each SNP individually along with a thorough analysis of their respective gene draws the same conclusion; there are no direct SNP or set of SNPs located within the direct subunit forming gene regions that explain the observed energetics variance.

**Table 5:** Six SNP missense variants were identified in *Mus musculus* strains that contain varying degrees of admixture.

			**No admixture**		**Nuclear admixture**		**Cytonuclear admixture**	
**SNP ID**	**Variant**	**Gene**	**RBF**	**CZECHII**	**MOLF**	**CALB**	**MOLG**	**SKIVE**
rs27071376	A	ATP5G1	T	T	T	A	T	T
rs27089394	A	ATP6V0A1	G	A	A	A	G	G
rs30941310	G	ATP6V0A4	C	G	G	C	G	G
rs32573927	C	ATP6V1D	T	C	C	T	C	C
rs13465886	A	Ndufs7	A	G	G	G	G	G
rs13480841	A	Uqcr10	A	G	A	G	A	G

*The SNPs were selected for investigation due to their contribution to the formation of the OXPHOS complexes. Displayed below are the SNP designation, the missense variant, the affected gene, and the allele present in each of the six mouse strains used for this study. Strains are separated based on degree of admixture present in their genomes. Alleles consistent with the missense variant of the protein are highlighted*.

Variant A of SNP rs27071376, present in CALB/RkJ, is part of Complex V. The gene in which this SNP is found plays a role in hydrogen ion transport, which is essential to drive chemiosmosis and create ATP (Rebhan et al., 1997). In theory, altering the protein product for this gene would have an impact on energy production, but this was not observed in the CALB/RkJ mice. However, ATP5G1 is one of three genes that code for the same protein in the complex (Rebhan et al., 1997), meaning loss of function in one gene is likely masked by the other two, thus limiting the impact of this variant, which explains why there was no phenotypic anomaly observed during the energetics analysis.

The next three SNPs, rs27089394, rs30941310, and rs32573927, all reside in genes forming the vacuolar ATPase that regulates mitochondrial matrix acidification through hydrogen ion transport (Rebhan et al., 1997). Although ATP6V0A4 has been linked to renal tubular acidosis, none of the specific SNPs indicated are implicated in the disease. Furthermore, all three genes have multiple known isoforms and alternatively spliced variants (Rebhan et al., 1997), suggesting that these variants likely have no negative impact on OXPHOS efficiency. This conclusion is consistent with the distribution of variants in each of these genes across the strains of mice in each admixture category. In order for a SNP of interest to have had a significant impact in our study, and therefore account for the observed results, we would expect a SNP variant to be present in either the cyto-nuclear admixed mouse strains or in the no admixture and nuclear admixture mouse strains, but not both; observed respiration rate differences cannot be a result of a variant common across all admixture groupings. None of the analyzed SNPs have such a distribution (Table 5). Likewise, rs13480841 is most likely not a significant factor in energy production levels because each allele variant is present in one of the two strains from each admixture group, not isolated to a single admixture group. The gene associated with rs13480841, Uqcr10, is a subunit of Complex III and is responsible for transferring electrons into the respiratory chain, a process which ultimately leads to proton transport (Schägger et al., 1995).

The most interesting finding in targeted SNPs is the presence of a variant allele for SNP rs13465886 in RBF/DnJ. This SNP is a part of the Ndufs7 gene, which codes for one of ~42 subunits of Complex I. Complex I is the first complex in the electron transport chain and inefficiencies in Complex I have been linked to neurological disorders (Trounce et al., 1994; Bénit et al., 2001). This SNP variant is also the only one to obtain a SIFT score, which indicates that the A allele carried in the RBF/DnJ strain is potentially deleterious. However, the potential effects of polymorphism at this locus were not evident during the energetic analysis of the heart and liver samples in these mice; RBF/DnJ mice did not have varying respiration rates. There are several possible explanations for this observation. First, the SIFT score was fairly low, 0.01 and it is not known that the presence of the A allele is deleterious, but rather that the reported effect is just presumed. A literature search did not provide evidence to support this prediction, as there was no study found which links this particular polymorphism to neurological disorders or any other disease or Complex I issue. Deficiency in Complex I can be caused by countless mutations, not limited to the polymorphism at rs13465886, and there is a possibility that this SNP has no discernable effect, especially in such a large complex. Additionally, even if there is selective pressure instigated by this polymorphism, Complex I is not the only entry point for the electron transport chain. Complex II, succinate dehydrogenase, will transfer hydrogens to an electron acceptor that can drive the rest of the pathway (Cecchini, 2003). Just as a change in ATP5G1 could be masked by another gene with overlapping function, Complex II activity could compensate for a minor decrease in Complex I activity. This sort of shift would not be noticed simply by measuring oxygen consumption rates. In order to confirm this hypothesis, additional tests must be performed in which the activity of each complex is assessed independently.

## Discussion

### Possible Consequences of Nuclear-Mitochondrial Admixture

We were unable to identify any underlying OXPHOS subunit-generating genetic causes for the observed energetic declines, which supports the hypothesis that admixture is creating maladaptive combinations of nuclear and mitochondrial proteins. If this study had identified a single SNP or a combination of SNPs that accounted for the shift in energetic levels in the highly admixed mice, then the discussion on cytonuclear incompatibility would be void because a mutation would explain these results. These results suggest that the problem is likely due to cytonuclear incompatibility, arising due to secondary contact and resultant subspecific hybrid matings that decouple the nuclear-mitochondrial ancestry. In order to fully validate our hypothesis, further testing and exhaustive analysis of additional gene regions would need to be performed, accounting for regulatory regions, transcription factors, and other genetic contributions to the OXPHOS pathway.

If admixture results in fluctuations in OXPHOS efficiency, as we theorize here, then it is expected that two individuals with the same nuclear genotypes will have different mitochondrial activity measurements if their mitochondria are of different lineages. This suggests that simply possessing a particular allele that causes shifts in energetics is not the sole effector of the decrease in activity. The interaction of that protein isoform with the subunit components, e.g., the varying mitochondrial alleles, may play a significant role. An example of such an incompatibility was demonstrated by Meiklejohn et al. in 2013 when they demonstrated that particular nuclear-encoded SNPs had no deleterious affects when paired with their concordant mitochondrial genome; however, when combined with discordant mtDNA, decreased fitness was observed (Meiklejohn et al., 2013) Although the mitochondrial genome was not individually assessed in this study, each strain was placed into a mitochondrial haplogroup based on a 19 SNP haplotype (Table 6). Three of the mouse strains share identical mitochondrial ancestry. Each of these three strains is a representative of a different admixture grouping (one with no admixture, one with nuclear admixture, and one with cytonuclear admixture), indicating their nuclear backgrounds vary significantly. This is evident by examining the six SNPs listed in Table 5. Each of the three strains have at least two of the six known SNP variants, yet only one variant, rs13480841, is present in two of these three strains. Variant nuclear background between the three strains sharing this same mitochondrial haplogroup is further confirmed when analyzing the subspecific genetic makeup of the nuclear DNA (Table 2). As previously mentioned, four of the six genes evaluated have functional redundancy in other genes, isoforms, or splicing variants. Each variant may itself be functional, but perhaps the different protein isoforms associate with other proteins differentially. It is probable that variants tolerated in the no admixture and nuclear admixture strains are detrimental in the cytonuclear admixture strains because they are not compatible with the proteins formed by the mitochondrially-derived subunits present. This may explain why the MOLG/DnJ strain of mice would have significantly lower basal respiration rates than the RBF/DnJ and CALB/RkJ strains, although they all share similar mitochondrial DNA haplotypes. In this case, the difference is likely due to the interaction of the proteins that come from that mtDNA with different versions of the collaborating proteins that are coded for by the nuclear genome.

**Table 6:** Mitochondrial haplotypes for different *Mus musculus* strains.

**Group**	**Strain**	**Mitochondrial haplogroup**																		
No Admixture	RBF/DnJ	C	T	C	C	C	T	C	T	T	C	C	A	T	A	G	A	G	A	T
	CZECHII/EiJ	T	T	C	T	T	C	T	T	C	T	T	G	C	T	A	A	A	G	T
Nuclear Admixture	MOLF/EiJ	T	T	T	T	T	C	T	T	C	T	T	G	C	T	A	A	A	G	T
	CALB/RkJ	C	T	C	C	C	T	C	T	T	C	C	A	T	A	G	A	G	A	T
Cytonuclear Admixture	MOLG/DnJ	C	T	C	C	C	T	C	T	T	C	C	A	T	A	G	A	G	A	T
	SKIVE/EiJ	C	T	C	C	T	C	N	T	T	C	C	G	T	A	G	A	G	A	T
Genomic Position:		2525	2615	2656	2786	2798	2814	2840	2918	3077	3140	3155	3220	3233	3329	3350	3362	3380	3392	3443

*The strains are separated by degree of admixture. The haplotypes for 19 mitochondrial SNPs were combined to create haplogroups^15^. Locations for each SNP along the mitochondrial genome are indicated at the bottom of the chart. Strains sharing the same haplogroup are highlighted*.

Furthermore, we analyzed the four strains of mice with the same predominant nuclear background together. In both the hepatic and the cardiac tissues, at least one cyto-nuclear admixed strain had respiration levels significantly lower than other strains. In the cardiac tissue, the two mice with no admixture and only nuclear admixture displayed no variation, although both had oxygen consumption levels significantly higher than those of the two cyto-nuclear admixed strains (Table 4). This confirms that the observed trends were not merely due to a main effect of a particular nuclear line. In fact, the distribution of nuclear ancestry between nuclear-admixed MOLF/EiJ and cyto-nuclear admixed MOLG/DnJ are nearly identical (Table 2, Supplementary Table 1, Supplementary Figures 3, 4), however, MOLF/EiJ pairs the predominantly nuclear *M. m. musculus* background with a *M. m. musculus* mitochondrial ancestry, while MOLG/DnJ has mitochondria of *M. m. domesticus* origin. The fact that the cardiac respiration rate of the cyto-nuclear admixed MOLG/DnJ strain was significantly lower than that of it's cyto-nuclear concordant counterpart MOLF/EiJ further supports our theory that these differences are in fact the result of cyto-nuclear incompatibility, in this case between the *M. m. musculus* nuclear genes and the *M. m. domesticus* mitochondrial ones.

Although the strains MOLG/DnJ and MOLF/EiJ have ancestral distributions similar across the nuclear genome, they do not have identical nuclear genomes. For this reason, it is impossible to definitively conclude there is not a strain-bias in our results. It is possible that variations present within the nuclear genomes between MOLG/DnJ and MOLF/EiJ contributed to the observed difference noted in basal respiration between the two. This once again highlights the need for an in-depth genetic comparison of all the strains in order to confirm our theory that cytonuclear incompatibility is the cause of the observed decline in basal respiration rates.

### The Presence of Nuclear Admixture Alone Did Not Reveal Significant Changes in Basal Respiration

Interestingly, incorporating nuclear genes from different subspecific backgrounds into a genome in which the mitochondrial and nuclear genomes are predominantly from the same phylogenetic origin, does not seem to affect energy production. In this study, “nuclear admixture” was defined as strains containing some variation in their nuclear genome, but the primary ancestral background is concordant with that of the mitochondrial genome. In the two strains evaluated of this category, over 75% of the nuclear background was consistent with the mitochondrial lineage across the entire genome, including the OXPHOS gene regions. According to Wallace's theory of aging, an accumulation of mitochondrial mutations throughout an organism's life leads to alterations in bioenergetics activity, which results in aging phenotypes (Wallace, 2005). The mechanism behind this theory is consistent with the mechanism proposed for this study. Mutations over the life-time create weaker protein interactions which decrease OXPHOS activity. The key is that the age-related phenotype is not present until enough mutations occur to surpass some currently unknown threshold in decreased subcomplex interaction. Perhaps, a < 75% uniformity does not exceed this threshold. The oxidative phosphorylation pathway is already susceptible to degeneration with time (Yen et al., 1989; Cooper et al., 1992; Ojaimi et al., 1999; Herbst et al., 2007), and studies suggest that any genetic disposition that shifts this already fragile system toward inefficiency could lead to aging and health disparity (Mishmar et al., 2003; Ruiz-Pesini et al., 2004). Perhaps nuclear admixture simply tips the scale and would result in reaching this threshold earlier in life, however, the ~12-week-old mice analyzed may not have accumulated a significant degree of mutations during the study period. Or perhaps, the genomes of these *Mus* subspecies are not sufficiently diverged to result in reduced the protein interactions.

Throughout this report, we have implied that the observed decrease in OXPHOS activity was a deleterious change. It is worth noting that, such a conclusion is merely speculative. With the exception of papers outlining various relationships between mammalian size and/or climate change to basal and maximal respiration rates (Scholander et al., 1950; Hayssen and Lacy, 1985; Koteja, 1987), a thorough literature search yielded no information on what the expected normal or critical respiration levels in *Mus musculus* may be. Additionally, there are no studies that provided oxygen consumption readings from the Seahorse Extracellular Flux Analyzer for closely related mammalian species allowing for direct comparison. Without further analysis, no concrete conclusion can be drawn about the impacts of the observed result. That being said, it would be naïve to believe a decrease in energy production, as was observed, would have no adverse effects on the organism, especially given the drop in basal respiration observed in two essential organs. Previous studies suggest that a decline in energy production contributes to the aging process (Pan, 2011; Tranah, 2011), susceptibility to age-related diseases (Chandrasekaran et al., 1996; Wang et al., 2009; López-Gallardo et al., 2011), and decreased lifespan (Correa et al., 2012), implying there is a direct correlation between OXPHOS efficiency and healthy longevity of an organism. When the findings of such studies are considered in light of the results of this experiment, one might suggest that highly admixed individuals have diminished healthy longevity levels as compared to individuals with concordant ancestral backgrounds. In fact, Latorre-Pellicer et al. demonstrated that of conplastic mice throughout the course of their lifetime presented distinctive differences in the overall health, including marked telomere shortening, mitochondrial dysfunction, and obesity (Latorre-Pellicer et al., 2016).

The scientific community now fully endorses the theory that coevolution between nuclear and mitochondrial genomes create highly efficient energy production pathways (Goodman, 1982; Grossman et al., 2004; Schmidt et al., 2005; Gershoni et al., 2010; Bar-Yaacov et al., 2012). It is well documented and understood that cytonuclear incompatibility can lead to inefficiencies in the OXPHOS pathway (Barrientos et al., 1998; Sackton et al., 2003; Ellison and Burton, 2006; Ellison et al., 2008; Niehuis et al., 2008), hybrid inviability (Takeda et al., 2000; Mihola et al., 2009), and decreased relative fitness (Latorre-Pellicer et al., 2016). Our study initiates the next step in the scientific process: understanding the mechanism and intricacies behind the potential energetics breakdown in admixed populations. We lay a foundation for future studies on potential admixture effects on coevolved metabolic systems and open up a deeper level of questions: What degree of admixture constitutes incompatibility resulting in deleterious effects? What is the long-term impact of nuclear admixture? What is the mechanism involved in correcting for incompatibility? In answering these questions, researchers will be able to predict potential health disparities resulting from the ever-changing human population boundaries. Advancements in knowledge at this level will eventually lead to ways of not only preventing prenatal and neonatal malformations, but also influence a more graceful aging process, possibly delaying or eliminating late-onset diseases, ultimately increasing both length and quality of the average life.

## Author Contributions

RZ and JP conceived the study. RZ conducted the experimental part. JP supervised the experimental part. RZ and JP wrote the manuscript.

### Conflict of Interest Statement

The authors declare that the research was conducted in the absence of any commercial or financial relationships that could be construed as a potential conflict of interest.
