# Supplementary figures and images for: Admixture Effects on Coevolved Metabolic Systems
Source: Front Genet. 2018 Dec 12;9:634. doi: 10.3389/fgene.2018.00634 (PMC6299042; doi:10.3389/fgene.2018.00634)

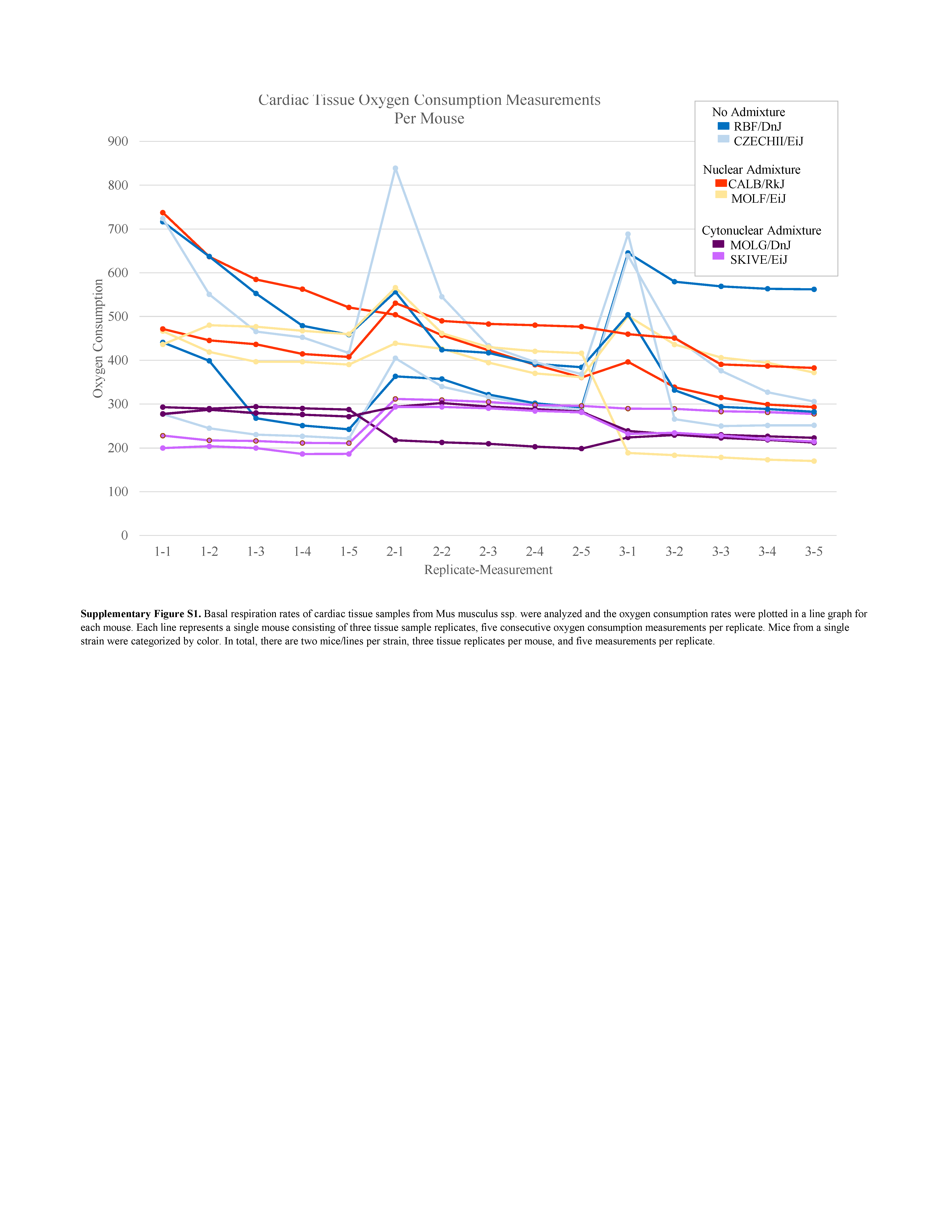

Supplement: Supplementary file 3 [file Image_1.TIFF]

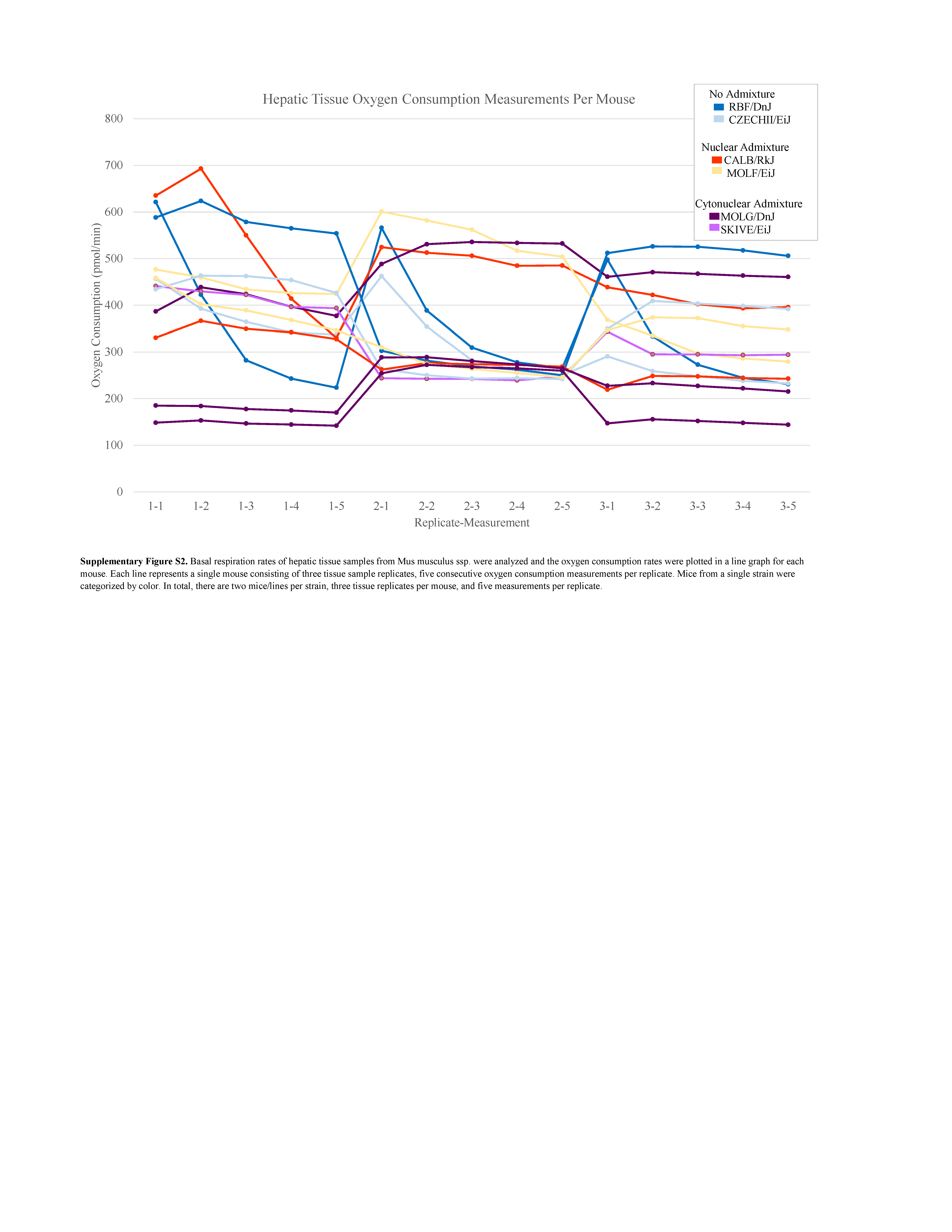

Supplement: Supplementary file 4 [file Image_2.TIFF]

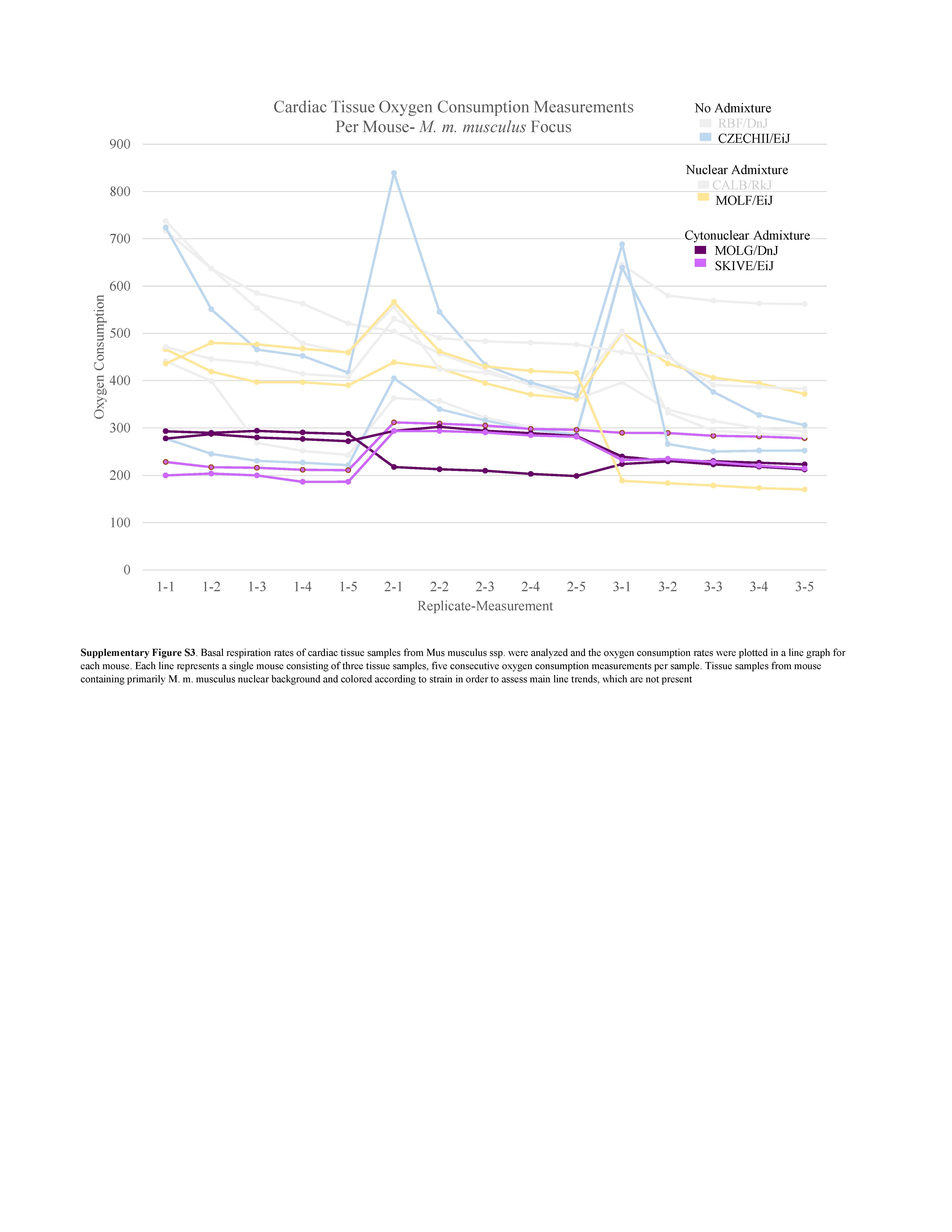

Supplement: Supplementary file 5 [file Image_3.TIFF]

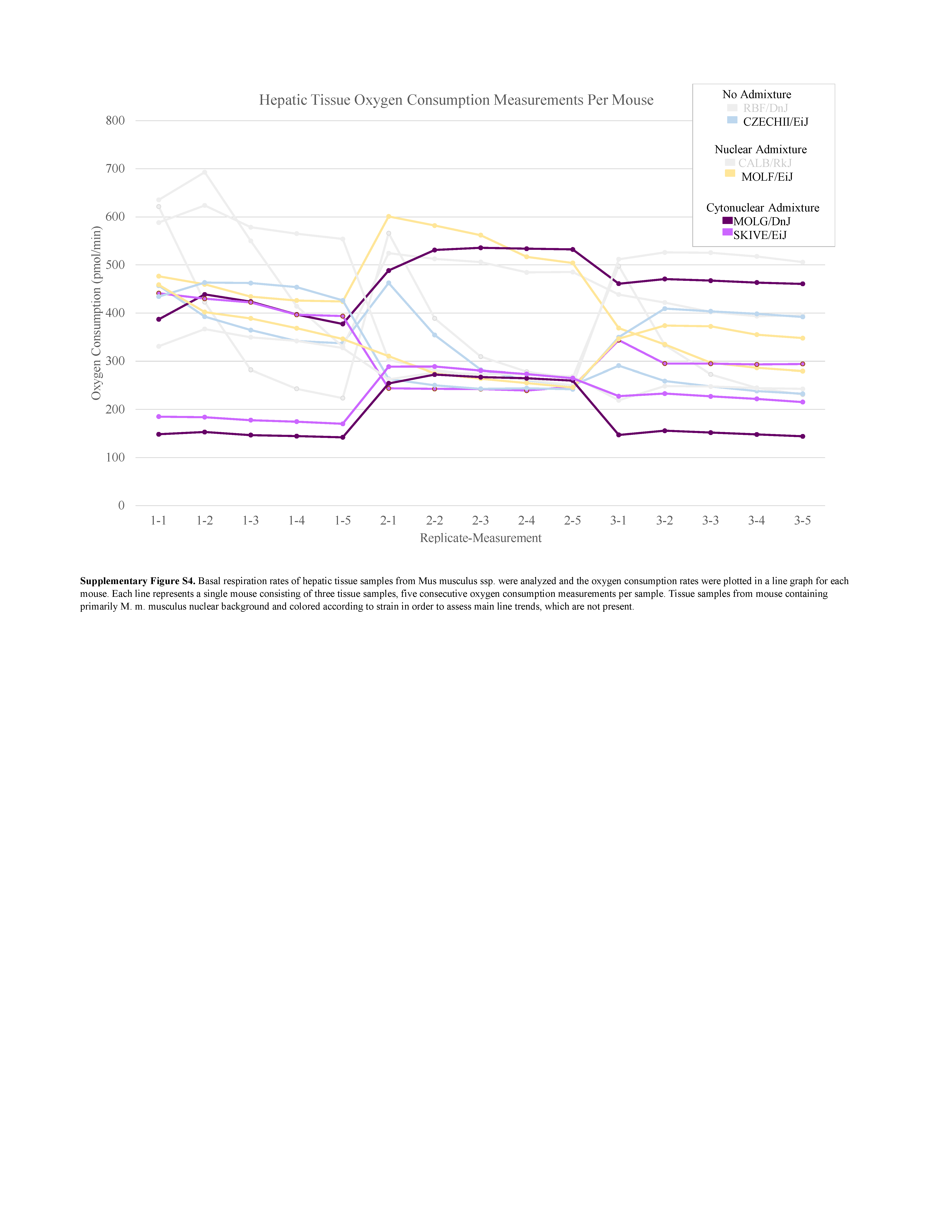

Supplement: Supplementary file 6 [file Image_4.TIFF]
